# Medical College Notes

**Published:** 1889-08

**Authors:** 


### Medical College Notes.

THE FACULTY OF THE COLLEGE OF PHYSICIANS AND SURGEONS OF BALTIMORE held a meeting last week to fill the vacancies created by the deaths of Professors John S. Lynch and Oscar J. Coskery, and the retirement of Professor A. B. Arnold, who has removed to San Francisco. Prof. Thos. S. Latimer was transferred to the chair of principles and practice of medicine and clinical medicine; Professor Chas. F. Bevan to the chair of principles and practice of surgery and clinical surgery; Professor J. W. Chambers to the chair of operative and clinical surgery, and Professor George H. Rohé to the chair of obstetrics and hygiene. Professor Thos. Opie will continue as professor of diseases of women, and dean of the Faculty. To fill vacancies created by these transfers, new professors were elected as follows: Professor Henry Sewall, of the University of Michigan, to the professorship of physiology; Dr. George J. Preston to the professorship of anatomy, with the diseases of the nervous system as a clinical branch of instruction. Dr. N. G. Keirle was elected as lecturer on legal medicine, in addition to his demonstrations in pathology; Dr. George Thomas as lecturer on diseases of the throat and chest; Dr. G. A. Liebig, Jr., of Johns Hopkins University, lecturer on medical electricity, and Dr. J. H. Branham, demonstrator of anatomy. Drs. L. F. Ankrim, Frank C. Bressler and F. G. Moyer were appointed assistant demonstrators, and Dr. R. G. Davis, prosector of anatomy. Professor Sewall, who comes here from the University of Michigan, is an old Baltimorean, and was for several years demonstrator of biology in Johns Hopkins University. All the other appointees are residents of this city. As an evidence of esteem on the part of his colleagues, Professor Arnold was elected emeritus professor of clinical medicine on his retirement.

---

THE MEDICO-CHIRURGICAL COLLEGE OF PHILADELPHIA.—The following changes in the faculty have been announced: Frank Woodbury, A. M., M. D., Honorary Professor of Clinical Medicine; William B. Atkinson, A. M., M. D., Honorary Professor of Sanitary Science and Pediatrics; John V. Shoemaker, A. M., M. D., Clinical Professor of Skin Diseases; James H. Anders, Ph. D., M. D., Professor of Hygiene, and Clinical Diseases of Children.

---

DR. JOHN PARMENTER has been appointed Lecturer on Anatomy in the Medical Department of the University of Buffalo.

